# Uncovering the Hidden Potential of Phytoene Production by the Fungus *Blakeslea trispora*

**DOI:** 10.3390/foods13182882

**Published:** 2024-09-11

**Authors:** Fani Th Mantzouridou, Elpida Sferopoulou, Panagiota Thanou

**Affiliations:** Laboratory of Food Chemistry and Technology, School of Chemistry, Aristotle University of Thessaloniki, 54124 Thessaloniki, Greece; sfairope@chem.auth.gr (E.S.); panagiwtathanou901@gmail.com (P.T.)

**Keywords:** phytoene, *Blakeslea trispora*, diphenylamine, 2-methyl imidazole, terbinafine, β-carotene, ergosterol, in vivo antioxidant activity

## Abstract

Phytoene is an uncommon linear carotene within the carotenoid group as it is colorless due to its short chromophore. Recent research constitutes a relatively new area which has emerged from phytoene’s importance as a major dietary carotenoid promoting health and appearance. Its resources point to the potential of biotechnological production systems. Our work has been designed to study the efficacy of two colored carotenoid biosynthesis inhibitors, diphenylamine and 2-methyl imidazole, and one sterol biosynthesis inhibitor, terbinafine, to modify the metabolic flux in mated cultures of *Blakeslea trispora* to achieve maximum phytoene production. Bioprocess kinetics optimized by response surface methodology and monitored by high-performance liquid chromatography revealed maximum phytoene content (5.02 mg/g dry biomass) and yield (203.91 mg/L culture medium) comparable or even higher than those reported for other potent phytoene microbial producers. The in vivo antioxidant activity of phytoene-rich carotenoid extract from fungal cells was also considered and discussed.

## 1. Introduction

Phytoene (PHY) (7,8,11,12,7,8,11,12-octahydro-ψ, ψ-carotene) is an uncommon linear carotene within the carotenoid group as it is colorless due to its short chromophore (3 conjugated double bonds). As a consequence of the latter structural feature, among other distinctive physicochemical properties, its maximum absorption occurs in the ultraviolet region (λmax of 286 nm in petroleum ether), far away from the wavelengths of 400–550 nm for colored carotenoids causing them to be yellow, orange or red [1]. Mainly due to its lack of color, which rendered its detection more difficult in the past, the field of PHY has been largely overlooked in carotenoid research. However, recent research constitutes a relatively new area which has emerged from PHY’s importance as a precursor of colored carotenoids but also as a major dietary carotenoid found in widely consumed foods such as tomatoes, oranges, red peppers, grapefruits and carrots, its bioavailability in humans and potential biological actions (antioxidant, anti-inflammatory and anti-carcinogenic activity or UVR-induced damage protection) promoting health and appearance [2,3]. 

Research on PHY has motivated the generation of novel products, such as extracts and powders rich in PHY used as functional ingredients and supplements for various sectors, including agro-food and cosmetics, and which are expected to extend from cosmeceuticals to functional foods/nutraceuticals [4]. These factors have boosted the demand for PHY, necessitating the development of novel methods that will enable the synthesis of large quantities of PHY. However, with regard to the occurrence of PHY and bioresources, its accumulation in high amounts is challenging considering its role as the starting molecule from which other primary and secondary carotenoids are formed. Its resources point to the potential of biotechnological production systems, including algae, yeasts and plant in vitro cultures. For example, few species of carotenogenic microalgae (*Chlorococcum* sp., *Dunaliella salina* and *Dunaliella bardawil*) have been shown to accumulate important amounts of PHY after herbicide treatment to inhibit pigment formation [5,6,7]. Several authors have driven the further development of PHY production through metabolic engineering strategies applied in the isoprenoid biosynthetic pathway in different biological systems, as described in a recent review [8]. Some examples of PHY-hyperproducing microbial strains are those of the methylotrophic bacterium *Paracoccus* sp. (up to 2 g/L of medium) [9], *D. salina* (up to ~1 mg/g biomass dry weight) [10], the red yeast *Xanthophyllomyces dendrorhous* (up to ~10 mg/g biomass dry weight) [11], the extremophilic bacterium *Deinococcus radiodurans* (up to ~1 mg/g biomass dry weight) [12] and the thermophilic archaea *Thermococcus kodakarensis* (up to ~2.5 mg/L of medium) [13]. Still, it can be claimed that the exploitation of microorganisms in the PHY production process is underutilized, especially when compared to the progress made for microbial production of colored carotenoids [14] and considering that the abovementioned organisms are still in the early stages of safe human usage.

The non-photosynthetic carotenogenic fungus *Blakeslea trispora* is important for the production of β-carotene (βC) and lycopene (LYC) due to its legal status and capacity to synthesize elevated levels of certain compounds that support elevated carotenoid output. Driven by the quest for sustainability, *B. trispora* has attracted the interest of researchers because of its capacity to convert a range of low-cost substrates and industrial waste streams into the high-value final product β-carotene, while also lowering manufacturing costs and moderating environmental pollution [15]. It thus appears to be a promising choice as a source of PHY for food and nutraceutical applications. In this fungus, carotenogenesis involves the co-culturing of mycelia of plus (+) and minus (−) mating types, which stimulates the metabolic flow towards the end product βC [16,17]. It is well-known that PHY is the first specific precursor of βC in the fungal cells. Two geranylgeranyl pyrophospate units derived from the mevalonate pathway are condensed to form PHY. Then, the successive addition of four double bonds to the PHY skeleton results in the synthesis of LYC. Finally, the rings at the ends of the molecule are formed. The two genes that encode the enzymes that are involved are the bifunctional PHY synthase-LYC-cyclase fusion gene with two individual domains (A and R domains of carRA, respectively) and the PHY desaturase (carB). To obtain LYC, the use of appropriate chemical or genetic means selectively suppress LYC cyclase activity in order to prevent the formation of βC in favor of LYC accumulation [18,19].

What is important is that, despite the research interest that *B. trispora* has attracted for enhanced production of βC and LYC, relevant studies on PHY production with this fungus practically do not exist. This has been previously assessed only to a very limited extent in studies on certain aspects of carotenogenesis in this fungus (i.e., effect of mating, regulatory mechanisms) [20,21,22]. Noticeably, these studies demonstrated that PHY has the potential to account for a very high percentage of carotenoids as a consequence of the occurrence of at least one rate-limiting enzyme (i.e., PHY synthase) and the use of PHY desaturase inhibitors (i.e., diphenylamine) in controlling flux to colored carotenoids. However, previous research can only be considered a first step for identifying the induction conditions necessary for optimizing PHY production by this fungus.

The biosynthetic pathway of sterols is involved in carotenoid synthesis by *B. trispora* through farnesyl diphosphate (FPP), which is the branch point for the two pathways. The first reaction of the pathway devoted solely to ergosterol (ERG) biosynthesis is catalyzed by squalene (SQU) synthase, which is also essential in directing intermediates to the sterol or non-sterol branches of this metabolic pathway. In this fungus, the mevalonate biosynthetic route to isoprenoid precursors leads mainly to the formation of ERG. However, very few studies have considered how to redirect the metabolic flux from sterols to carotenoids in living cells of *B. trispora* [23,24,25]. 

To address the gaps in research outlined above, our work has been designed to study the efficacy of two colored carotenoid biosynthesis inhibitors, diphenylamine (DPA) and 2-methyl imidazole (MI), and one sterol biosynthesis inhibitor, terbinafine hydrochloride (TH), to modify the metabolic flux in mated cultures of *B. trispora* to achieve maximum PHY production. TH, a member of the allylamine group of antimycotics, acts by inhibiting SQU epoxidase to oxidize SQU to 2,3-oxidoSQU [23]. DPA blocks the sequence of desaturation reactions by inhibiting PHY synthase [22]. MI prevents LYC cyclization by inhibiting LYC cyclase [16]. The bioprocess was undertaken, combining the action of the three chemical regulators at different levels along with the cultural stimulants in terms of the (+) and (−) strain ratio in the inoculum, the addition time (h) of inhibitors and fermentation time according to a central composite statistical design (CCD) and response surface methodology (RSM). Chromatographic methods were applied to monitor the carotenoids and sterols in fungal cells. To our knowledge, this is the first study of the combined effect of TH, DPA and MI on the kinetics of PHY formation. The antioxidant activity tested in vivo toward the protection of *Saccharomyces cerevisiae* cells from H_2_O_2_-induced oxidative stress [26] were also considered and discussed. 

## 2. Materials and Methods

### 2.1. Microorganisms

The microorganisms used for fermentation experiments were *B. trispora* ATCC 14271, mating type (+), and *B. trispora* ATCC 14272, mating type (−). The strains were grown separately on potato dextrose agar (PDA) petri dishes at 26 °C for 5 d to prepare the spore inoculums. Stock cultures were stored as spore suspensions at −80 °C in cryotubes containing 20% glycerol solution. The laboratory yeast strain that was used for the in vivo antioxidant activity assay was the wild-type *Saccharomyces cerevisiae* EGY48 (ura3 trp1 his3 6LexA-operator-LEU2) generously provided by Dr. Antonios Makris (Inst. of Applied Biosciences (INEB) of the National Center for Research and Technological Development (CERTH), Thessaloniki, Greece). The yeast strain was maintained at 4 °C on yeast extract peptone dextrose (YPD) agar (1% *w/v* yeast extract, 2% *w/v* peptone, d-glucose, and 2% *w/v* agar) slants.

### 2.2. Standards, Reagents and Solvents

15-*cis* PHY (20% in oil) was a gift of Lycored Ltb (Beer Sheva, Israel) to our laboratory. All-*trans* LYC (purity ≥ 95%) was from Extrasynthese (Genay, France). All-*trans* βC (purity 97%) was purchased from Merck (Darmstadt, Germany). SQU (purity 98%), lanosterol (purity ≥ 93%) and zymosterol (purity > 99%) were from Sigma-Aldrich Co. (St. Louis, MO, USA), and ERG (purity > 98%) was from BDH Biochemicals Ltd. (Poole, UK). PDA was from LabM (Lancashire, UK). The culture medium contained D-glucose monohydrate (Duchefa Biochemie, Haarlem, The Netherlands), corn steep liquor (Sigma-Aldrich Co.), casein acid hydrolysate (LabM), yeast extract (Neogen, Lansing, MI, USA), L-asparagine (Panreac Quimica S.A., Barcelona, Spain), KH_2_PO_4_ (Merck), MgSO_4_·7H_2_O (Panreac Quimica S.A.), thiamine-HCl (Alfa-Aesar, Karlsruhe, Germany), Span 20 (Alfa-Aesar), and Tween 80 (TCI Europe, Zwijndrecht, Belgium). DPA was from Fluka AG (Buchs, Switzerland), TH was from TCI Europe and MI was from Sigma-Aldrich Co. High-performance liquid chromatography (HPLC)-grade methanol, acetonitrile, propanol-2 and water were from Chem-Lab (Zedelgem, Belgium). All of the other common reagents and solvents were of the appropriate purity from various suppliers.

### 2.3. Apparatus

Extraction of carotenoids from fungal cells was assisted by ultrasonic treatment using a Sonopuls Ultrasonic Homogenizer (Bandelin electronics GmbH & Co., Berlin, Germany) consisting of an ultrasonic generator (GM 2070, 70 W, 20 kHz), an ultrasonic transducer (UW 2070) and a standard probe (MS 72 Titanium microtip, 2 mm). Reversed-phase HPLC (RP-HPLC) analysis of carotenoids was performed isocratically using a solvent delivery system consisting of a LC-20AD pump (Shimadzu, Kyoto, Japan), a S5300 autosampler (Sykam, Eresing, Germany) with a 100 µL fixed loop and a 505 LC column oven (Rigas Laboratories, Thessaloniki, Greece). The chromatograph was coupled to a SPD-M40 diode array multiple-wavelength detector (Shimadzu). Chromatographic data were processed with the software LabSolutions Single PDA (Shimadzu). RP-HPLC analysis of SQ and sterols was performed on a Shimadzu Nexera X2 UHPLC System (Shimadzu Corporation, Kyoto, Japan), equipped with a LC-30AD pump, a SIL-30AC autosampler (50 μL loop), a CTO-20AC column oven and a UV/visible diode array SPD-M20A detector (temperature controlled semi-micro flow cell of 2.5 μL). Chromatographic data were processed with the software Lab Solution (version 5.86, Shimadzu). Inoculated flasks were placed in a shaking incubator (KS 4000 ic control, IKA, Staufen, Germany). An Orion Star A111 pH probe (Thermo Fisher Scientific, Darmstadt, Germany) was used to measure the pH of the culture medium. The cellular-based antioxidant activity assay was conducted by using a UV-1601 spectrophotometer (Shimadzu) and UV-probe 2.33 data handling software. 

### 2.4. Preparation of Inocula 

The fungal strains were grown separately on PDA at 26 °C for 3–5 days for heavy sporulation and used for the inoculation of the culture medium with appropriate volumes of spore suspensions of each strain containing ~1.0 × 10^6^ spores/mL.

### 2.5. Fermentation Conditions

The batch fermentation was conducted in 250 mL conical flasks at a filling volume of 50 mL of the culture medium with the following composition (g/L): 50.0 (glucose), 80.0 (CSL), 2.0 (casein acid hydrolysate), 1.0 (yeast extract), 2.0 (l-asparagine), 1.5 (KH_2_PO_4_), 0.5 (MgSO_4_·7H_2_O) and 0.005 (thiamine-HCl). Span 20 (10.0 g/L) and Tween 80 (1.0 g/L) were added to achieve dispersed growth of *B. trispora*. The shaking speed was 180 rpm and the flasks were incubated at 26 °C. Fermentation experiments were carried out in triplicate.

### 2.6. Experimental Design

Fifty-three experiments were set according to a CCD for the study of six factors, namely DPA (*X*_1_), MI (*X*_2_) and TH (*X*_3_) levels of addition (mg/L of culture medium), addition time of inhibitors (*X*_4_) (h), fermentation time (*X*_5_) (h) and (+)/(−) strain ratio (*X*_6_), each at five experimental levels. DPA and TH were dissolved in ethanol and MI in distilled water. All of the above solutions were sterilized by passing through a 0.45 μm membrane filter (Schleicher Schnell, Dassel, Germany) before inoculation. The levels of the *X*j factor are coded as follows: −a, −1, 0, +1, and +a, where a = 2^n/4^, n = number of variables, and −1, +1 and 0 correspond to the low, high and middle levels of each factor. The actual levels of each factor were calculated according to the formula

(1) $$\mathrm{Coded}\;\mathrm{value}=\frac{actual\;level-\frac{high\;level\;+\;low\;level}{2}}{\frac{high\;level\;-\;low\;level}{2}}$$

where −1, +1 were 11.8, 29.0 mg/L for *X*_1_; 102.0, 250.0 mg/L for *X*_2_; 26.5, 65.0 mg/L for *X*_3_; 19.6, 48.0 h for *X*_4_; 76.0, 168.0 h for *X*_5_; and 0.2, 0.35 for *X*_6_.

The 53 runs were set using Minitab Statistical Software Version 22.1.0.0 (free trial, Minitab, Inc., State College, PA, USA) (Table S1). The design had nine of the factorial points at the center of the design replicated for the estimation of error. Polynomial response surfaces were fitted to response variables, namely PHY content (mg/g of dry biomass) (*Y*_PHY_), LYC content (mg/g of dry biomass) (*Y*_LYC_), SQU content (mg/g of dry biomass) (*Y*_SQU_) and ERG content (mg/g of dry biomass) (*Y*_ERG_). Statistical analysis of the experimental data was performed by RSM using the same software. Initially, the second-order polynomial model was fitted to each response giving an equation of the form *Y* = *β*_0_ + *β*_1_*Χ*_1_ + *β*_2_*Χ*_2_ + *β*_3_*Χ*_3_ + *β*_4_*Χ*_4_ + *β*_5_*Χ*_5_ + *β*_6_*Χ*_6_ + *β*_11_*Χ*_11_^2^ + *β*_22_*Χ*_22_^2^ + *β*_33_*Χ*_33_^2^ + *β*_44_*Χ*_44_^2^ + *β*_55_*Χ*_55_^2^ + *β*_66_*Χ*_66_^2^ + *β*_12_*Χ*_1_*Χ*_2_ + *β*_13_*Χ*_1_*Χ*_3_ + *β*_14_*Χ*_1_*Χ*_4_ + *β*_15_*Χ*_1_*Χ*_5_ + *β*_16_*Χ*_1_*Χ*_6_ + *β*_23_*Χ*_2_*Χ*_3_ + *β*_24_*Χ*_2_*Χ*_4_ + *β*_25_*Χ*_2_*Χ*_5_ + *β*_26_*Χ*_2_*Χ*_6_ + *β*_12_*Χ*_1_*Χ*_2_ + *β*_34_*Χ*_3_*Χ*_4_ + *β*_35_*Χ*_3_*Χ*_5_ + *β*_3_*Χ*_3_*Χ*_6_ + *β*_45_*Χ*_4_*Χ*_5_ + *β*_46_*Χ*_4_*Χ*_6_ + *β*_56_*Χ*_5_*Χ*_6_ (Equation (1)), where *Y* is the dependent variable (response); *X*_1_, *X*_2_, *X*_3_, *X*_4_, *X*_5_ and *X*_6_ represent the levels of the coded factors, and *β*_0_, *β*_1_, …, *β*_56_ represent the estimated coefficients, *β*_0_ being a scaling constant. The quality of the fit of the model was evaluated by the coefficients of determination (*R*^2^), the significance of each parameter through F-test (calculated *p*-value) and the lack of fit of the model. Coefficients with a *p*-value lower than 0.05 were considered significant. Where possible, the model was simplified by omission of statistically non-significant terms. Optimization of the fitted polynomial for *Y*_PHY_ (mg/g dry biomass) was performed using the same software. The combination of factor optimal values resulting in a maximum *Y*_PHY_ value was verified by conducting a simulation experiment (Test I) to investigate the kinetics of carotenoid and sterol contents in *B. trispora* cells for a time period from 0 to 164 h, in triplicate. Results were compared with a control experiment without the addition of inhibitors (Test II) and model predictions.

### 2.7. Determination of Biomass Dry Weight 

At the end of the bioprocess, culture medium was centrifuged (4500× *g*, 10 min, 4 °C) in order to separate the biomass from the liquid broth. The cells were washed by mixing directly with 20 mL of 85/15 isopropanol/water azeotrope, following vortex and centrifugation. The alcoholic resuspension thus prepared was filtered, using a Buchner funnel, so that the solids content in the filtrate liquid was practically zero. The resulting purified wet mycelium contained the carotenoids produced in fermentation. Wet biomass was vacuum dried at a temperature of 45 °C for 120–200 min to reach a final moisture ≤ 8% [27]. Total biomass was determined from dry matter (g/L of culture medium). The repeatability of the method was satisfactory (CV% = 2.26, *n* = 3). Dry biomass was kept at −40 °C until further analysis.

### 2.8. Extraction and RP-HPLC of Carotenoids

The dry biomass was ground manually, using pestle and mortar, in order to obtain a fine powder permitting solvent extraction. The carotenoids were extracted from fungal cells by ultrasonication with a standard probe for 5 min and at a specific amplitude (97%). With the aid of an ice bath, the sample temperature did not exceed 20 °C. A portion of 0.1 g of dry biomass was accurately weighed and mixed with 10 mL of ethyl acetate into the processing tube. The organic phase was separated by centrifugation at 4500× g for 10 min (4 °C). The same procedure was repeated until full depigmentation of fungal cells (2–4 extraction cycles). This process occurred in duplicate for each sample, and then the two extracts were mixed. The repeatability of extraction was satisfactory (CV% = 5.56, *n* = 3). All treatments were accomplished away from light exposure. The cell-free extract was used to determine the profile and content of carotenoids by RP-HPLC. 

The analysis of carotenoids by RP-HPLC was performed using the method described by Pollmann et al. [11], with slight modifications. Crude extracts were first dissolved in a small amount of tetrahydrofuran and then in acetonitrile/methanol/2-propanol (40:50:10, by volume). All samples were filtered through a 0.45 μm membrane filter before chromatographic analysis. HPLC analysis was performed on a reversed phase Nucleosil C18 (250 × 4 mm, i.d. 5 μm) column with acetonitrile/methanol/2-propanol (40:50:10, by volume) as mobile phase with a flow rate of 2 mL/min at 30 °C, and the injection volume was 50 µL. Peak identification was achieved by comparing the retention time with that of authentic standards and confirmed by spiking and comparison of spectral data. For neurosporene, γ-carotene, ζ-carotene, β-zeacarotene and 7,8-dihydro-β-carotene, their identification was solely based on elution order and spectral data in the visible region with regards to published information [28,29,30]. Quantification of PHY, LYC and βC was carried out at 286, 472 and 452 nm with the aid of standard curves calculated by linear regression analysis. Analysis of samples was carried out in duplicate (CV% = 0.5, 0.78 and 0.62, respectively, for a 3 mg/L PHY, a 4 mg/L LYC and a 3 mg/L βC standard solution; *n* = 5). 

### 2.9. Extraction and RP-HPLC Analysis of SQU and Sterols

SQU and sterols were extracted after ethanolic pyrocatechol saponification as follows: Dried cells (0.1 g) were resuspended in 8 g of KOH and 32 mL of 60% (*v*/*v*) ethanolic pyrocatechol solution (0.07%, *w*/*v*) and saponified at 80 ± 2 °C in water bath for 2 h. The unsaponified matter (UM) was extracted by the addition of 10 mL of hexane. The phases were separated by centrifugation at 3500 rpm for 10 min. The top hexane layer was removed to a clean tube, and the residue was extracted one more time with hexane. Emulsions, if present, can be dispersed by the addition of 0.5 mL of methanol. The hexane fractions were combined, a certain amount of extract was received and finally the solvent was removed under nitrogen gas. Repeatability of extraction was satisfactory (CV% = 8.4 and 5.4, respectively, for SQU and ERG, *n* = 3). Crude extracts were kept at −18 °C until further analysis.

The UM was dissolved in methanol. All samples were filtered through a 0.45 μm membrane filter just before HPLC. Separation of SQU and sterols was achieved on a reversed phase Accucore C_18_ column (100 × 4.6 mm, i.d. 2.6 μm) maintained at 30 °C. The elution solvent was methanol/water (98:2, *v*/*v*), the flow rate was set at 0.8 mL/min and the injection volume was 10 μL. Detection and quantification of SQU and ERG were at 208 and 280 nm, respectively. Peaks were identified by comparison of retention time with that of authentic standards and confirmed by comparison of spectral data. Quantification of SQU and ERG was accomplished with the aid of standard curves calculated by linear regression analysis. Analysis of samples was carried out in duplicate (CV% = 2.8 and 1.5, respectively, for 2 ppm SQU and 1 ppm ERG standard solutions; *n* = 3). Ergosterol derivative (ergosta-5,7,22,24(28)-tetraen-3β-ol, 22,23-dihydroergosterol) identification was solely based on elution order and spectral data in the UV region with regard to published information [31,32].

### 2.10. In Vivo Antioxidant Activity Assay

The cellular-based antioxidant activity assay was performed according to Wu et al. [26], with modifications. The wild-type yeast strain *S. cerevisiae* EGY48 was used. Cells were cultured overnight in 30 mL YPD containing single standard PHY (100 μΜ) and PHY-enriched fungal extract (100 μM PHY) to induce an antioxidant response. PHY standard stock solution and extract were prepared in acetone and sterilized by passing through a 0.45 μm membrane filter (Schleicher Schnell, Dassel, Germany) before inoculation. Yeast cultures were then centrifuged at 1000× *g* for 5 min and cells first washed with acetone and then with sterile distilled water. Washed cells were transferred into fresh YPD medium (at an OD_600_ value of 0.2) containing H_2_O_2_ (4 mM) and incubated for 2 h at 28 °C on a rotary shaker at 120 rpm (oxidant-treated cells) against the untreated control. Cell viability was determined by measuring the OD_600_ of the cultures at the end of incubation. Survival percentages were normalized to control samples (100% viability). All assays were carried out in triplicate.

### 2.11. Statistical Analysis 

Statistical comparison of the mean values was performed by one-way ANOVA, followed by the multiple Duncan test (*p* < 0.05 confidence level) using SPSS 29.0 software (SPSS Inc., Chicago, IL, USA).

## 3. Results and Discussion

The manipulation of metabolic regulation by chemical means can be utilized to modify the metabolic flux in *B. trispora* cells to produce desirable chemicals with superior yields and productivity. The concentration ranges of regulators should be set so as to exert high effectiveness without affecting the fungal growth ability. In this study, selection of the level of inhibitors to improve PHY production by *B. trispora* with regards to yield, selectivity and safety aspects was based on relevant literature data [16,23,33]. In this view, DPA, MI and TH were tested at levels up to 40 mg/L, 350 mg/L and 90 mg/L, respectively (Table S1). A CCD was used to select the experimental conditions under which the study of the effects of DPA (*X*_1_), MI (*X*_2_) and TH (*X*_3_) levels of addition (mg/L of culture medium), addition time of inhibitors (*X*_4_) (h), fermentation time (*X*_5_) (h), and (+)/(−) strain ratio (*X*_6_) on PHY (*Y*_PHY_), LYC (*Y*_LYC_), SQU (*Y*_SQU_) and ERG (*Y*_ERG_) content (mg/g dry biomass) takes place for the mated cultures of *B. trispora* strains. Dry biomass weight was satisfactory for a typical fermentation process ranging from 22.56–31.28 g/L of culture medium [16]. In addition, in the absence of 2-methyl imidazole, βC content reached 1.12 mg/g dry biomass, while its presence was detected in traces (≤0.09 mg/g dry biomass). The responses shown in Table 1 are discussed in Section 3.1, Section 3.2, Section 3.3, Section 3.4 and Section 3.5. 

Visualization of the effects of the independent factors (*X*_1_, *X*_2_, *X*_3_, *X*_4_, *X*_5_ and *X*_6_) on the responses is provided through the response surface plots shown in Section 3.1, Section 3.2, Section 3.3 and Section 3.4. The statistical models fitted to the data for each response allowed assessment of the interactions among the factors using a reduced number of experiments. The models, in terms of coded (−1, +1) and actual factor levels (see Experimental Design, Table S1) fitted for each of the response variables are shown in Table 2 (Equations (2)–(9)). 

ANOVA of Models 1–4 revealed significance of regression (*p* < 0.05) and non-significant lack-of-fit (*p* > 0.05), while coefficient of determination (R^2^) values were from 0.775–0.882, which indicates that more than 77% of the variability of the responses is explained by the models. Statistical details and a description of the model fitting are provided in the Supporting Information (data in Tables S2–S6). 

### 3.1. Effect on Y_PHY_

**Figure 1:**
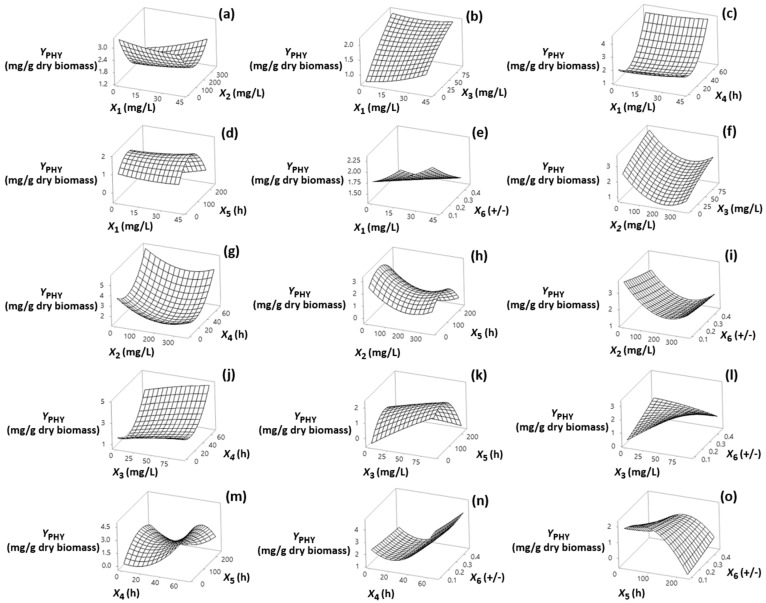
(**a**–**o**) Three-dimensional surface plots for phytoene content (*Y*_PHY_, mg/g dry biomass) as a function of diphenylamine (*X*_1_), 2-methyl imidazole (*X*_2_) and terbinafine (*X*_3_) levels of addition (mg/L of culture medium), addition time of inhibitors (*X*_4_) (h), fermentation time (*X*_5_) (h) and (+)/(−) strain ratio (*X*_6_). In all cases, the rest of the factors were kept constant at their middle levels (20.4 mg/L, 175 mg/L, 45.8 mg/L, 33.8 h, 122 h and 0.28 +/−, respectively).

By comparing data in Figure 1, panels a, b and f among inhibitors, the most marked increase in *Y*_PHY_ was seen with regard to the increase of TH levels of addition, especially at values approaching its middle level (45.8 mg/L). 

This finding may be explained by the fact that TH activity has been linked with the induction of the rate-limiting enzyme of the mevalonate pathway 3-hydroxy-3-methylglutaryl coenzyme A (HMG-CoA) reductase via inhibiting SQU epoxidase to oxidize SQU to 2,3-oxidoSQU [23]. Moreover, it may act in a way that is similar to the way in which naftifine, another allylamine, acts, contributing to de-pigmentation in *Rhodotorula mucilaginosa* through down-regulation on expression of PHY desaturase gene CAR1 [34]. Nevertheless, exposing fungal cells to TH at higher than the middle level of 45.8 mg/L resulted in a less marked impact on *Y*_PHY_ (Figure 1b,f). *Y*_PHY_ values became substantially higher when the addition time of TH reached its highest value (67.6 h) (Figure 1j). This result ties well with previous research in which successful inhibition of the activity of crucial enzymes involved in the pathways of ERG biosynthesis was associated with the decline phase of cell growth that coincides with the synthesis of carotenoids in fungal cells [23]. However, in the ongoing cultivation, a strong decrease in *Y*_PHY_ was observed (Figure 1d,h,k,m), which may be due to the reversible inhibition effect of TH on SQU epoxidase activity due to its loose interactions with the binding domains of the enzyme, resulting in TH easy removal over time [32].The data in Figure 1a,f revealed a strong negative impact of MI on *Y*_PHY_, which was reversed above 176 mg/L of the inhibitor (middle level of addition). On the other hand, although non-significant, there was a small positive linear and quadratic effect of DPA on *Y*_PHY_ (Figure 1a–c, Table S3). Also, optimal for *Y*_PHY_ is the predominance of carotenogenic (−) *B. trispora* strain in inoculums (Figure 1e,i,l,n,o). It is possible that a considerably larger carotene producing capacity of the (–) strain as compared with the (+) strain, and therefore its preferential development, is important for active carotenogenesis [35]. Interestingly, a positive interconnection of TH with the prevalence of the carotenogenous (–) strain in the mated culture was found, since these factors showed a mutually positive effect on *Y*_PHY_ (Figure 1l, Table S3). 

### 3.2. Effect on Y_LYC_

**Figure 2:**
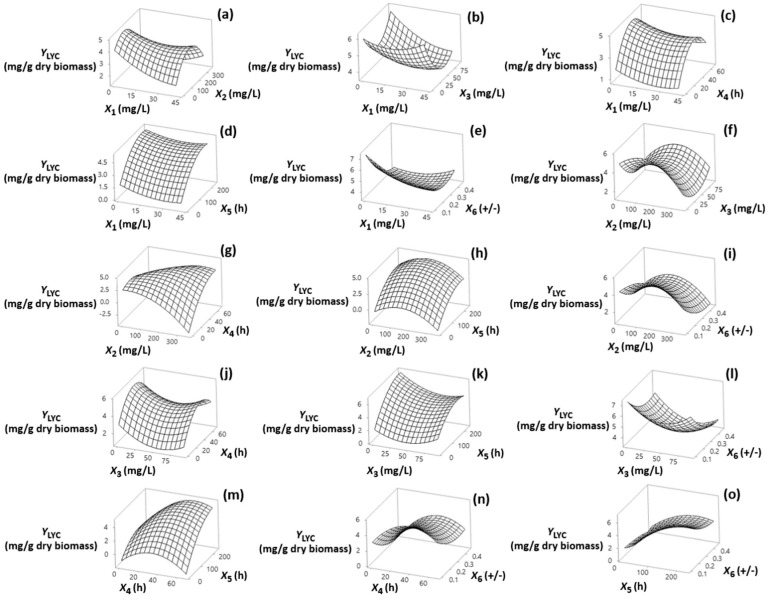
(**a**–**o**) Three-dimensional surface plots for lycopene content (*Y*_LYC_, mg/g dry biomass) as a function of diphenylamine (*X*_1_), 2-methyl imidazole (*X*_2_), and terbinafine (*X*_3_) levels of addition (mg/L of culture medium), addition time of inhibitors (*X*_4_) (h), fermentation time (*X*_5_) (h) and (+)/(−) strain ratio (*X*_6_). In all cases, the rest of the factors were kept constant at their middle levels (20.4 mg/L, 175 mg/L, 45.8 mg/L, 33.8 h, 122 h and 0.28 +/−, respectively).

Data in Figure 2a,b revealed decreasing *Y*_LYC_ with respect to increasing DPA and TH up to the middle exposure inhibitor levels of fungal cells (20.4 mg/L, and 45.8 mg/L, respectively), although not significant (Table S4). 

The results of the Sun et al. study [23] indicated that the use of high doses instead of low doses of the TH inhibitor reduces LYC content in treated *B. trispora* cells. By comparing the patterns of LYC and PHY content in treated cells, *Y*_LYC_ varied inversely with *Y*_PHY_, indicating that these compounds act by preventing the synthesis of colored carotenoids in fungal cells in favor of PHY accumulation. Regarding the effect of MI, Figure 2f exposes the strong negative quadratic effect of MI on *Y*_LYC_. This means that, reversible to the case of *Y*_PHY_, a further increase of MI values above their middle level caused a strong decrease in *Y*_LYC_. A similar conclusion was reached by Pegklidou et al. [16] by using an inhibitor concentration range of 10–200 mg/L. The negative quadratic effect of MI on *Y*_LYC_ was less pronounced by increasing the inhibitor addition time up to the middle level (Figure 2g). In the *Y*_LYC_ response surface plots (Figure 2d,h,k,m,o), a trend toward higher *Y*_LYC_ values was noted as the fermentation time approached its middle value (100 h), while this variable exhibited the opposite effect on *Y*_PHY_. Moreover, as shown in Figure 2e,i,l,n,o, higher levels of LYC accumulated at low values of (+)/(−) strain ratios, similar to findings found for *Y*_PHY_. Thus, it appears that the use of (+)/(−) ratios near 1(+)/7(−) controls cell growth and favors secondary metabolism and more specifically carotenogenesis. This is consistent with what has been found in previous studies of the carotenogenesis inhibition by MI to increase lycopene synthesis in fungal cells [16].

### 3.3. Effect on Y_SQU_

**Figure 3:**
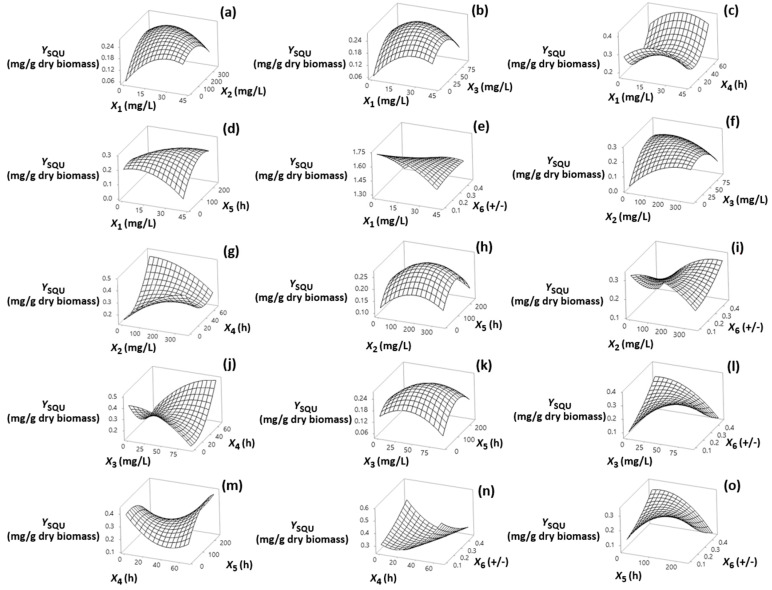
(**a**–**o**) Three-dimensional surface plots for squalene content (*Y*_SQU_, mg/g dry biomass) as a function of diphenylamine (*X*_1_), 2-methyl imidazole (*X*_2_) and terbinafine (*X*_3_) levels of addition (mg/L of culture medium), addition time of inhibitors (*X*_4_) (h), fermentation time (*X*_5_) (h) and (+)/(−) strain ratio (*X*_6_). In all cases, the rest of the factors were kept constant at their middle levels (20.4 mg/L, 175 mg/L, 45.8 mg/L, 33.8 h, 122 h and 0.28 +/−, respectively).

Data in Figure 3a,b,f revealed increasing *Y*_SQU_ with respect to increasing DPA, MI, and TH up to the middle exposure inhibitor levels of fungal cells (20.4 mg/L, 176 mg/L and 45.8 mg/L, respectively); but these effects were negligible compared with the larger effects of MI (negative) and TH (positive) on *Y*_PHY_ (Table S5). 

In the same figures, the characteristic curvatures of the response surfaces illustrate a negative quadratic effect of inhibitors on *Y*_SQU,_ which were marginally significant for DPA and MI (*p* = 0.059 and 0.080, respectively), and significant for TH (*p* = 0.031) (Table S5). These findings suggest that the dose-dependent inhibitory activity of inhibitors is related to their specific mechanism of action. The results in Figure 3j indicate that the increase of inhibitor addition time significantly heightens TH performance, particularly by showing stronger positive impact as the addition time approaches its maximum value (67.6 h). But, a significant negative interaction effect was noted for MI and its addition time on *Y*_SQU_. Higher levels of *Y*_SQU_ accumulated at low-to-medium fermentation times (Figure 3d,h,k,m,o). This phenomenon is reversed at higher addition times of MI (Figure 3m). 

### 3.4. Effect on Y_ERG_

**Figure 4:**
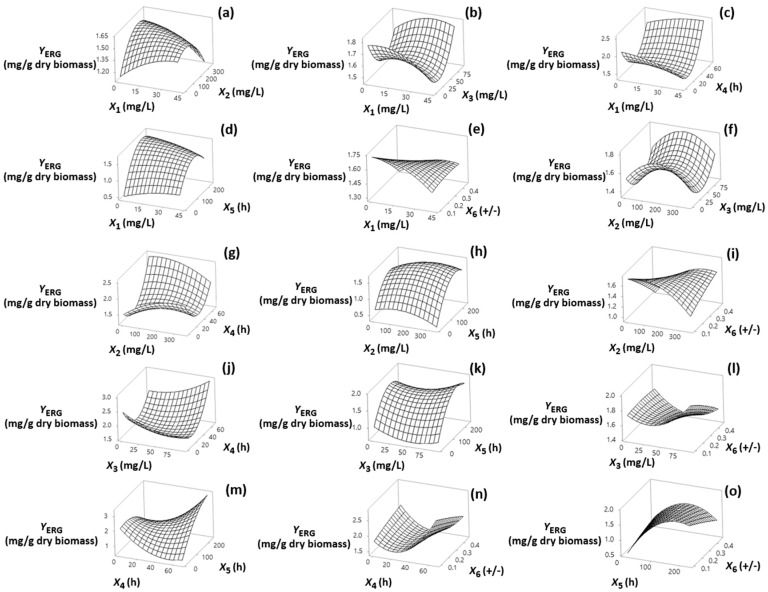
(**a**–**o**) Three-dimensional surface plots for ergosterol content (*Y*_ERG_, mg/g dry biomass) as a function of diphenylamine (*X*_1_), 2-methyl imidazole (*X*_2_) and terbinafine (*X*_3_) levels of addition (mg/L of culture medium), addition time of inhibitors (*X*_4_) (h), fermentation time (*X*_5_) (h) and (+)/(−) strain ratio (*X*_6_). In all cases, the rest of the factors were kept constant at their middle levels (20.4 mg/L, 175 mg/L, 45.8 mg/L, 33.8 h, 122 h and 0.28 +/−, respectively).

As shown in Figure 4m, where the most significant factors for *Y*_ERG_ are displayed, it becomes obvious that its value increased in fermentation time near the middle value (122 h). 

This can be attributed to the fungus’s ability to synthesize ERG during the early stages of its growth as an important component of its cell membrane. Specifically, this main sterol regulates membrane structure fluidity, permeability, and membrane-bound enzyme activities, as well as substance transportation [23]. Also, *Y*_ERG_ increased as the addition time of inhibitor approached its maximum value (Figure 4c,g,j,m,n). Inhibiting ERG synthesis during the early stages of the bioprocess by chemical means can be pivotal for fungal growth [36]. Noticeably, the strong and positive interaction effect of *X*_4_ and *X*_5_ indicates that the longer the addition time of inhibitor and fermentation time the higher the *Y*_ERG_ (Figure 4m, Table S6). Despite the non-significant effect, it was observed that the interaction *X*_3_*X*_5_ was positive for *Y*_ERG_ (Figure 4k). Thus, in an ongoing cultivation, the increase of *Y*_ERG_, which was followed by the concomitant decrease in *Y*_SQU_ (Figure 3k), is due to the reversible inhibition effect of this agent on SQU epoxidase activity [32]. 

### 3.5. Optimum Conditions for Y_PHY_

As the fitted model for Equation (3) in Table 2 provides a good approximation of the experimental conditions, this was employed to predict optimum values of the process variables for maximum *Y*_PHY_. The terbinafine (*X*_3_) level of addition (of 91.53 mg/L of culture medium), inhibitor addition time (*X*_4_) (of 67.57 h), fermentation time (*X*_5_) (of 125 h) and (+)/(−) strain ratio (*X*_6_) (of 0.1), but without the addition of diphenylamine (*X*_1_) and 2-methyl imidazole (*X*_2_), can be recommended as optimum for maximum *Y*_PHY_. 

### 3.6. Validation of the Optimized Conditions for Y_PHY_

**Figure 5:**
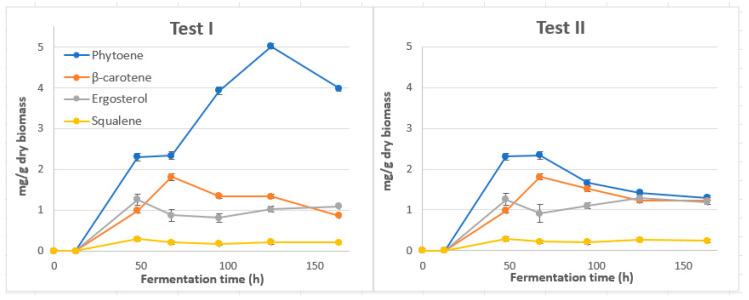
Time course of synthesis of phytoene, β-carotene, ergosterol and squalene in *B. trispora* cells cultivated under the predicted combination of factor optimal values resulting in maximum *Y*_PHY_ (Test I) and in the control experiment without the addition of terbinafine (Test II).

The combination of factor optimal values resulting in the maximum *Y*_PHY_ value was validated by conducting a simulation experiment (Test I). Apart from evaluating PHY content, we put special effort into examining whether and how the bioprocess conditions may affect the carotenoid and sterol pattern present in fungal cells for a time period from 0 to 164 h. Results were compared with a control experiment without the addition of terbinafine (Test II) (Figure 5).

**Figure 6:**
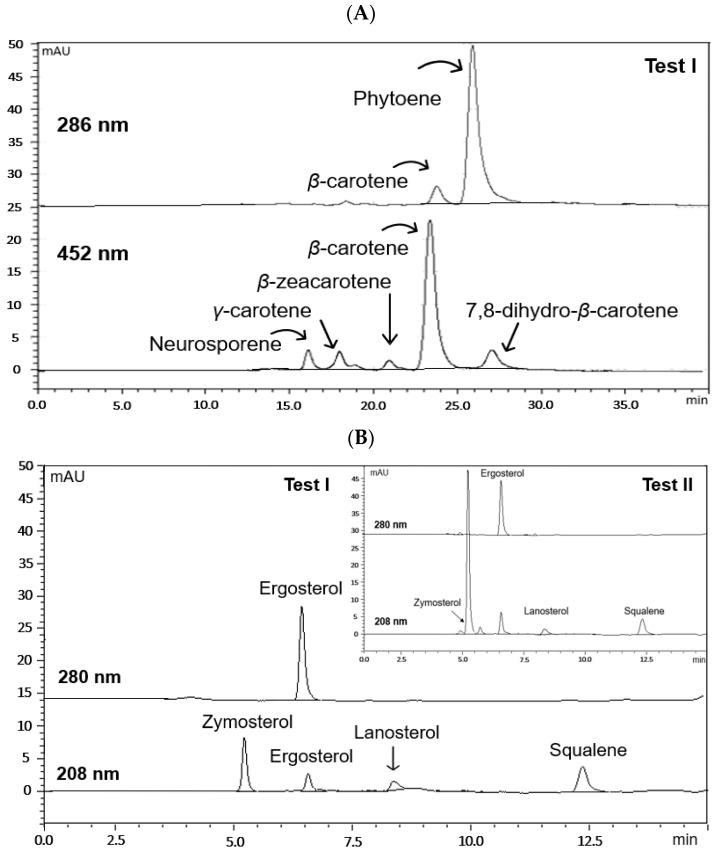
HPLC chromatograms of the phytoene-rich carotenoid extract (**A**) and the UM of cellular lipids (**B**) from TH-treated *B. trispora* cells (fermentation time, 125 h) under the predicted combination of factor optimal values resulting in maximum *Y*_PHY_ (Test I) and in the control experiment without the addition of terbinafine (Test II).

The chromatographic profiles obtained from the RP-HPLC-DAD analysis of the various carotenoid-rich extracts recorded at 286 nm and 452 nm were qualitatively similar. At this stage, existing literature data about the elution order of carotenoids on the reverse-phase C18 column were carefully scrutinized [29,30] to extract valuable information. Spectral characteristics and t_R_ values were also compared with those of available standard compounds. As a result, it was confirmed that they presented six peaks related to carotenoids (Table S7). In particular, at 286 nm, the major peak was identified as PHY (peak 6). The RP-HPLC profile at 452 nm shows, in addition to the substrate PHY, the accumulation of βC (peak 5) and the intermediates in the biosynthetic chain from PHY to βC, neurosporene (peak 1), γ-carotene (peak 2), ζ-carotene (peak 3), β-zeacarotene (peak 4) and 7,8-dihydro-β-carotene (peak 7) (Figure 6A).

RP-HPLC profiles of the UM of cellular lipids from the treated cells with TH, recorded at 208 nm and 280 nm, were qualitatively similar to those found for the untreated cells. Different intermediates (ergosta-5,7,22,24-(28)-tetraen-3β-ol (peak 1), zymosterol (peak 2), 22,23-dihydroergosterol (peak 4), lanosterol (peak 5) and squalene (peak 6), along with ERG (peak 3), were formed in fungal cells (Table S8, Figure 6B)). 

Differences were mainly quantitative. Maximum PHY content and yield (5.02 mg/g dry biomass and 203.91 mg/L culture medium, respectively) were achieved within 125 h of cultivation under the optimum experimental conditions set in Test I. In the absence of TH (Test II), PHY reached its maximum value (2.34 mg/g) at 67.57 h and then decreased as fermentation time increased. In both series of tests, βC synthesis showed a gradual increase until 67.57 h, where it reached its maximum value (1.81 mg/g dry biomass), and then decreased. 

Under the optimum cultivation conditions for PHY production, ERG and SQU reached their maximum values (1.26 and 0.29 mg/g dry biomass, respectively) at 48 h. Of particular importance is the fact that ERG and SQU synthesis was not significantly affected by the addition of TH to the culture medium, as a slightly higher maximum content of both compounds in fungal cells was observed in the absence of the inhibitor (Figure 5). This was not the case for zymosterol, the content of which was reduced by almost 85% in the presence of TH (Test I) compared to that reported for untreated cells (Test II), although not at the expense of ERG content (Figure 6). Our results support previous evidence related to the increase in enzyme activity of (HMG-CoA) reductase in response to a selective depletion of endogenous sterols that make refluxing of more FPP intermediate, a common precursor of sterol and *β*-carotene synthesis, towards the branched carotenoid biosynthesis pathway more likely [24,37]. To our knowledge, this is the first attempt to expose *B. trispora* cells to an increased level of TH (91.5 mg/L) on the kinetics of PHY accumulation. Looking for the toxicological aspects of the various inhibitors applied in this study, the toxicity of TH (LD_50_ value) [38] seems to be lower than that of DPA [39] and MI [40]. This information provides a good indication for the possible reasons of selecting TH over a wide range of other possible inhibitors for PHY accumulation in *B. trispora* cells. 

To our knowledge, maximum PHY content per g of dry biomass achieved in this study (5.02 mg/g dry biomass and 203.91 mg/L culture medium, respectively) was the highest reported by mated fermentation of *B. trispora*. In terms of process selectivity, PHY accounted for more than 60% of total carotenoid content of fungal cells. According to the literature data, PHY content of fungal cells ranges from 0.36 (without inhibitors) to 2.91 mg/g dry biomass, assisted by diphenylamine [22]. Breitenbach et al. [20] investigated PHY production in wild type (1.09 mg/g dry biomass) and carotenogenic mutants of *B. trispora* (1.48 mg/g dry biomass). Similar PHY levels were reported for wild type and mutant strains of *Phycomyces blakesleeanus* under DPA treatment [41,42]. Levels of PHY achieved in this study are at least 20-fold higher than those reported for various plant-based foods like tomatoes and derivatives [3]. Also, the yield of phytoene production (203.91 mg/L culture medium) is analogous to that reported for norflurazon-treated *D. bardawil* cells in biphasic systems (300 mg/L culture medium), a microalgae known for its ability to synthesize high levels of the colourless carotenoid [6]. It should also be pointed out that PHY content obtained in TH-treated *B. trispora* cells of the present study is directly comparable to that of engineered *D. salina* (up to ~1 mg/g biomass dry weight) [10], the extremophilic bacterium *D. radiodurans* (up to ~1 mg/g biomass dry weight) [12], the thermophilic archaea *T. kodakarensis* (up to ~2.5 mg/L of medium) [13] and the red yeast *X. dendrorhous* (up to ~10 mg/g biomass dry weight) [11]. Looking for novel resources, the methylotrophic bacterium *Paracoccus* sp. (up to 2 g/L of medium) [9] has been proposed. However, it should be stressed that all of the above potent microbial factories for this functional lipid production are not yet organisms with a history of safe human use, so that to get legal status, a petition to interested parties is required. Getting this permission is a tedious, expensive and time-consuming process.These findings seem encouraging toward the manipulation of metabolic regulation by the inhibitor TH, an effective tool to enhance PHY accumulation in *B. trispora* cells.

### 3.7. Cytoprotective Effect of Phytoene-Rich Extract in H_2_O_2_-Stressed S. cerevisiae Cells

The antioxidant potential of microbial PHY has not yet been studied, despite the few studies reporting on the microbial synthesis of PHY. In the current study, the cytoprotective effect of the PHY-rich carotenoid extract from *B. trispora* biomass cultivated under the experimental conditions of Test I was examined in *S. cerevisiae* BY4741 cells upon exposure to 4 mM H_2_O_2_ for 2 h. According to the data in Figure 5, yeast cells were sensitive to the oxidizing agent, and only 20% of the yeast population was able to survive the induced oxidative stress. Upon treatment of yeast cells with the PHY-rich carotenoid extract containing 100 µM PHY, the strong increase in the recovery of the surviving cells (93%) above the oxidant-induced death, even more than that for the yeast cells treated with the standard solution of the same PHY concentration (80%), indicated a strong antioxidant activity of the phytoene-rich carotenoid extract [3]. 

## 4. Conclusions

The data of the present study, along with the fact that *B. trispora* is important for the production of carotenoids due to its legal status and capacity to produce elevated levels of PHY, comparable to other microbial sources that have been proposed as potent PHY producers, suggest the favorability of using the fungus to produce this uncommon linear carotene. Our findings seem encouraging toward the direction of scaling up in bioreactors, driven by a quest for sustainability that should be carried out together with a respective provision of process economics.

## Figures and Tables

**Table 1:** Experimental response values for phytoene (*Y*_PHY_), lycopene (*Y*_LYC_), squalene (*Y*_SQU_) and ergosterol (*Y*_ERG_) content (mg/g dry biomass).

	Responses (mg/g Dry Biomass)			
Run	*Y*_PHY_	*Y*_LYC_	*Y*_SQU_	*Y*_ERG_
1	0.98 ± 0.04	5.19 ± 0.12	0.18 ± 0.04	1.65 ± 0.09
2	4.46 ± 0.18	0.72 ± 0.04	0.47 ± 0.12	2.74 ± 0.07
3	1.67 ± 0.08	3.03 ± 0.11	0.27 ± 0.04	1.30 ± 0.04
4	1.88 ± 0.09	3.09 ± 0.09	0.24 ± 0.03	1.49 ± 0.12
5	1.49 ± 0.04	4.54 ± 0.08	0.27 ± 0.18	1.45 ± 0.18
6	3.62 ± 0.12	3.24 ± 0.09	0.17 ± 0.07	1.18 ± 0.05
7	2.49 ± 0,09	3.28 ± 0.13	0.21 ± 0.06	1.10 ± 0.04
8	1.59 ± 0.07	5.78 ± 0.09	0.32 ± 0.04	2.16 ± 0.12
9	1.12 ± 0.06	3.81 ± 0.05	0.20 ± 0.13	1.56 ± 0.09
10	1.50 ± 0.04	3.43 ± 0.06	0.31 ± 0.04	1.64 ± 0.03
11	2.14 ± 0.06	2.28 ± 0.03	0.30 ± 0.06	1.51 ± 0.07
12	1.60 ± 0.06	3.63 ± 0.07	0.31 ± 0.12	1.86 ± 0.06
13	1.43 ± 0.07	3.51 ± 0.07	0.23 ± 0.18	1.43 ± 0.08
14	0.73 ± 0.03	3.40 ± 0.06	0.19 ± 0.04	1.63 ± 0.13
15	1.30 ± 0.05	3.46 ± 0.09	0.13 ± 0.06	1.09 ± 0.08
16	1.67 ± 0.06	1.38 ± 0.05	0.30 ± 0.09	1.74 ± 0.07
17	1.02 ± 0.05	0.77 ± 0.03	0.34 ± 0.05	1.63 ± 0.04
18	1.63 ± 0.06	3.57 ± 0.10	0.22 ± 0.10	1.70 ± 0.18
19	1.48 ± 0.04	3.78 ± 0.09	0.25 ± 0.18	1.62 ± 0.13
20	1.85 ± 0.08	3.60 ± 0.07	0.21 ± 0.12	1.95 ± 0.06
21	1.37 ± 0.05	4.66 ± 0.09	0.17 ± 0.05	1.56 ± 0.10
22	2.41 ± 0.09	3.29 ± 0.11	0.19 ± 0.08	1.40 ± 0.18
23	1.49 ± 0.04	4.87 ± 0.08	0.22 ± 0.12	1.58 ± 0.07
24	2.41 ± 0.08	3.04 ± 0.07	0.26 ± 0.06	1.37 ± 0.09
25	2.58 ± 0.09	2.94 ± 0.06	0.20 ± 0.06	1.47 ± 0.03
26	2.08 ± 0.08	3.36 ± 0.08	0.21 ± 0.10	1.49 ± 0.04
27	1.22 ± 0.05	4.60 ± 0.04	0.18 ± 0.05	1.78 ± 0.09
28	1.17 ± 0.05	2.20 ± 0.08	0.18 ± 0.04	1.61 ± 0.12
29	0.00 ± 0.00	0.00 ± 0.00	0.00 ± 0.00	0.00 ± 0.00
30	2.20 ± 0.08	5.13 ± 0.13	0.47 ± 0.07	2.43 ± 0.05
31	3.28 ± 0.09	0.07 ± 0.03	0.17 ± 0.08	1.22 ± 0.12
32	1.67 ± 0.06	2.46 ± 0.05	0.25 ± 0.08	1.57 ± 0.07
33	1.53 ± 0.07	3.61 ± 0.09	0.27 ± 0.04	1.62 ± 0.18
34	1.38 ± 0.05	2.42 ± 0.07	0.25 ± 0.18	1.51 ± 0.06
35	1.95 ± 0.08	6.25 ± 0.11	0.23 ± 0.13	1.85 ± 0.05
36	2.36 ± 0.08	3.44 ± 0.05	0.24 ± 0.08	1.39 ± 0.04
37	2.25 ± 0.07	4.52 ± 0.06	0.18 ± 0.04	1.60 ± 0.18
38	1.64 ± 0.06	5.28 ± 0.08	0.20 ± 0.06	1.45 ± 0.06
39	1.81 ± 0.08	3.73 ± 0.09	0.28 ± 0.05	1.63 ± 0.05
40	1.32 ± 0.04	5.85 ± 0.05	0.19 ± 0.07	1.73 ± 0.08
41	2.77 ± 0.09	2.68 ± 0.06	0.30 ± 0.06	1.52 ± 0.09
42	3.88 ± 0.08	3.57 ± 0.04	0.32 ± 0.09	1.67 ± 0.08
43	2.68 ± 0.09	3.14 ± 0.05	0.20 ± 0.04	1.52 ± 0.18
44	1.72 ± 0.06	4.85 ± 0.09	0.21 ± 0.18	1.73 ± 0.06
45	1.62 ± 0.06	2.89 ± 0.05	0.22 ± 0.04	1.23 ± 0.05
46	0.86 ± 0.04	1.69 ± 0.03	0.26 ± 0.06	1.66 ± 0.09
47	1.30 ± 0.03	2.95 ± 0.05	0.36 ± 0.12	1.32 ± 0.08
48	1.68 ± 0.07	3.93 ± 0.08	0.23 ± 0.18	1.88 ± 0.07
49	1.01 ± 0.05	0.95 ± 0.04	0.26 ± 0.06	1.80 ± 0.10
50	0.52 ± 0.03	3.65 ± 0.06	0.22 ± 0.09	1.93 ± 0.03
51	1.75 ± 0.07	5.43 ± 0.08	0.34 ± 0.09	1.65 ± 0.13
52	1.51 ± 0.05	4.84 ± 0.09	0.23 ± 0.13	1.61 ± 0.05
53	1.57 ± 0.06	3.39 ± 0.04	0.30 ± 0.06	1.62 ± 0.08

**Table 2:** Model Equations for Prediction of the Optimum Response Values of Phytoene Content (*Y*_PHY_), Lycopene content (*Y*_LYC_), Squalene Content (*Y*_SQU_), and Ergosterol Content (*Y*_ERG_).

		Polynomial Equations	
Model	Response	Coded Value of Factors	Actual Value of Factors
1	*Y*_PHY_ (mg/g dry biomass)	*Y*_PHY_ = 1.610 − 0.2405*X*_2_ + 0.2355*X*_3_ + 0.4592*X*_4_ − 0.2156*X*_5_ − 0.1280*X*_6_ + 0.1691*X*_2_^2^ + 0.2733*X*_4_^2^ − 0.2225*X*_5_^2^ − 0.1551*X*_3_*X*_6_ − 0.2068*X*_4_*X*_5_ (2)	*Y*_PHY_ = 0.69 − 0.01762*X*_2_ + 0.0608*X*_3_ − 0.0231*X*_4_ + 0.0266*X*_5_ − 1.20*X*_6_ + 0.000031*X*_2_^2^ + 0.001355*X*_4_^2^ − 0.000105*X*_5_^2^ − 0.1075*X*_3_*X*_6_ − 0.000317*X*_4_*X*_5_ (3)
2	*Y*_LYC_ (mg/g dry biomass)	*Y*_LYC_ = 3.851 − 0.232*Χ*_2_ + 0.344*X*_4_ + 0.777*X*_5_ − 0.463*X*_6_ − 0.328*X*_2_^2^ − 0.366*X*_4_^2^ + 0.487*X*_2_*X*_4_ (4)	*Y*_LYC_ = 6.35 − 0.0043*Χ*_2_ + 0.0170*X*_4_ + 0.0453*X*_5_ − 18.5*X*_6_ − 0.000060*X*_2_^2^ − 0.001813*X*_4_^2^ + 0.000463*X*_2_*X*_4_ (5)
3	*Y*_SQU_ (mg/g dry biomass)	*Y*_SQU_ = 0.2669 + 0.00511Χ_3_ + 0.01141Χ_4_ − 0.00424Χ_5_ − 0.01316Χ_3_^2^ + 0.02043Χ_4_^2^ − 0.01492Χ_5_^2^ − 0.02219Χ_2_Χ_4_ + 0.02656Χ_3_Χ_4_ + 0.00656Χ_3_Χ_5_ − 0.02281Χ_3_Χ_6_ + 0.01844Χ_4_Χ_5_ − 0.01719Χ_4_Χ_6_ − 0.01844Χ_5_Χ_6_ (6)	*Y*_SQU_ = −0.027 + 0.00606Χ_3_ − 0.00604Χ_4_ + 0.00100Χ_5_ − 0.000036Χ_3_^2^ + 0.000101Χ_4_^2^ − 0.000007Χ_5_^2^ − 0.000021Χ_2_Χ_4_ + 0.000097Χ_3_Χ_4_ + 0.000007Χ_3_Χ_5_ – 0.01580Χ_3_Χ_6_ + 0.000028Χ_4_Χ_5_ − 0.01614Χ_4_Χ_6_ − 0.00534Χ_5_Χ_6_ (7)
4	*Y*_ERG_ (mg/g dry biomass)	*Y*_ERG_ = 1.5826 + 0.0995Χ_4_ + 0.1522Χ_5_ + 0.1127Χ_4_^2^ − 0.0879Χ_5_^2^ + 0.1703Χ_4_Χ_5_ (8)	*Y*_ERG_ = 1.84 − 0.0573Χ_4_ + 0.00760Χ_5_ + 0.000559Χ_4_^2^ − 0.000042Χ_5_^2^ + 0.000261Χ_4_Χ_5_ (9)

*X*_1_, *X*_2_, *X*_3_, *X*_4_, *X*_5_ and *X*_6_ are the coded (Equations (2), (4) and (6)) or actual (Equations (3), (5) and (7)) values of factors presented in Experimental Design. *Y*_PHY_*, Y*_LYC_*, Y*_SQU_ and *Y*_ERG_ are expressed in mg/g dry biomass.

## Data Availability

The original contributions presented in the study are included in the article/Supplementary Material, further inquiries can be directed to the corresponding author.

## Supplementary Materials

The following supporting information can be downloaded at: https://www.mdpi.com/article/10.3390/foods13182882/s1, Table S1. Experimental Design for Five-Level-Six-Factor Central Composite Design; Table S2. Analysis of variance of *Y*_PHY_, *Y*_LYC_, *Y*_SQU_ and *Y*_ERG_ obtained using the RSM model; Table S3. Estimated regression coefficients and significance (*p* values) for phytoene content (PHY) after analysis using coded values of factors; Table S4. Estimated regression coefficients and significance (*p* values) for lycopene content (LYC) after analysis using coded values of factors; Table S5. Estimated regression coefficients and significance (*p* values) for squalene content (SQU) after analysis using coded values of factors; Table S6. Estimated regression coefficients and significance (*p* values) for ergosterol content (ERG) after analysis using coded values of factors; Table S7. Carotenoid elution order on Nucleosil C18 column, retention time (RT), relative retention time (RRT), spectral characteristics of peaks (λmax) and possible identity based on the comparison with standard compounds and literature data [28,29,30]. Chromatographic data represent the carotenoid-rich extract from TH-treated *B. trispora* cells (fermentation time, 125 h); Table S8. Sterols elution order on Accucore C18 column, retention time (RT), relative retention time (RRT), spectral characteristics of peaks (λmax) and possible identity based on the comparison with standard compounds and literature data [32]. Chromatographic data represent the UM of cellular lipids from the TH-treated *B. trispora* cells (fermentation time, 125 h).

## References

[1] Tsimidou M.Z., Mantzouridou F.T., Nenadis N. (2023). Minor bioactive lipids. Adv. Food Nutr. Res..

[2] Meléndez-Martínez A.J., Mapelli-Brahm P. (2021). The undercover colorless carotenoids phytoene and phytofluene: Importance in agro-food and health in the Green Deal era and possibilities for innovation. Trends Food Sci. Technol..

[3] Meléndez-Martínez A.J., Mapelli-Brahm P., Stinco C.M. (2018). The colourless carotenoids phytoene and phytofluene: From dietary sources to their usefulness for the functional foods and nutricosmetics industries. J. Food Compos. Anal..

[4] Meléndez-Martínez A.J., Stinco C.M., Mapelli-Brahm P. (2019). Skin carotenoids in public health and nutricosmetics: The emerging roles and applications of the UV radiation-absorbing colourless carotenoids phytoene and phytofluene. Nutrients.

[5] Laje K., Seger M., Dungan B., Cooke P., Polle J., Holguin F.O. (2019). Phytoene αccumulation in the novel microalga *Chlorococcum* sp. using the pigment synthesis inhibitor Fluridone. Mar. Drugs.

[6] León R., Vila M., Hernánz D., Vílchez C. (2005). Production of phytoene by herbicide-treated microalgae *Dunaliella bardawil* in two-phase systems. Biotechnol. Bioeng..

[7] Xu Y., Harvey P.J. (2020). Phytoene and phytofluene overproduction by *Dunaliella salina* using the mitosis inhibitor chlorpropham. Algal Res..

[8] Miras-Moreno B., Pedreño M.Á., Romero L.A. (2019). Bioactivity and bioavailability of phytoene and strategies to improve its production. Phytochem. Rev..

[9] De La Fuente Moreno J.F., Rodriguez J.L., Costa S.Μ., Estrella De Castro P.J., Barredo Fuente A., López Ortiz J.L. (2009). Method for the Production of Phytoene and/or Phytofluene or Mixtures of Carotenoids Having a High Content of Same.

[10] Srinivasan R., Babu S., Gothandam K.M. (2017). Accumulation of phytoene, a colorless carotenoid by inhibition of phytoene desaturase (PDS) gene in *Dunaliella salina* V-101. Bioresour. Technol..

[11] Pollmann H., Breitenbach J., Sandmann G. (2017). Development of *Xanthophyllomyces dendrorhous* as a production system for the colorless carotene phytoene. J. Biotechnol..

[12] Jeong S.W., Kang C.K., Choi Y.J. (2018). Metabolic Engineering of *Deinococcus radiodurans* for the production of phytoene. J. Microbiol. Biotechnol..

[13] Fuke T., Sato T., Jha S., Tansengco M.L., Atomi H. (2018). Phytoene production utilizing the isoprenoid biosynthesis capacity of *Thermococcus kodakarensis*. Extremophiles.

[14] Ashokkumar V., Flora G., Sevanan M., Sripriya R., Chen W.H., Park J.H., Kumar G. (2023). Technological advances in the production of carotenoids and their applications–A critical review. Bioresour. Technol..

[15] Papadaki E., Mantzouridou F.T. (2021). Natural β-carotene production by *Blakeslea trispora* cultivated in Spanish-style green olive processing wastewaters. Foods.

[16] Pegklidou K., Mantzouridou F., Tsimidou M.Z. (2008). Lycopene production using *Blakeslea trispora* in the presence of 2-methyl imidazole: Yield, selectivity, and safety aspects. J. Agric. Food Chem..

[17] Wang Y., Wang Y., Wang Y., Chen X., Liu C., Zhang M., Liu K., Zhao Y., Li Z. (2021). Untargeted global metabolomic analysis reveals the mechanism of tripropylamine-enhanced lycopene accumulation in *Blakeslea trispora*. Front. Bioeng. Biotechnol..

[18] Mantzouridou F., Tsimidou M.Z. (2008). Lycopene formation in *Blakeslea trispora*. Chemical aspects of a bioprocess. Trends Food Sci. Technol..

[19] Wang Y.H., Zhang R.R., Yin Y., Tan G.F., Wang G.L., Liu H., Zhuang J., Zhang J., Zhuang F.Y., Xiong A.S. (2023). Advances in engineering the production of the natural red pigment lycopene: A systematic review from a biotechnology perspective. J. Adv. Res..

[20] Breitenbach J., Fraser P.D., Sandmann G. (2012). Carotenoid synthesis and phytoene synthase activity during mating of *Blakeslea trispora*. Phytochemistry.

[21] Hsu W.J., Yokoyama H., Coggins C.W. (1972). Carotenoid biosynthesis in *Blakeslea trispora*. Phytochemistry.

[22] Thomas D.M., Goodwin T.W. (1967). Studies on carotenogenesis observations-I.: General observations on synthesis in mated and unmated strains. Phytochemistry.

[23] Sun Y., Yuan Q.P., Vriesekoop F. (2007). Effect of two ergosterol biosynthesis inhibitors on lycopene production by *Blakeslea trispora*. Process Biochem..

[24] Tang Q., Li Y., Yuan Q.P. (2008). Effects of an ergosterol synthesis inhibitor on gene transcription of terpenoid biosynthesis in *Blakeslea trispora*. Curr. Microbiol..

[25] Wang H.B., He F., Lu M.B., Zhao C.F., Xiong L., Yu L.J. (2014). High-quality lycopene overaccumulation via inhibition of γ-carotene and egosterol biosyntheses in *Blakeslea trispora*. J. Funct. Foods.

[26] Wu M.J., O’Doherty P.J., Fernandez H.R., Lyons V., Rogers P.J., Dawes I.W., Higgins V.J. (2011). An antioxidant screening assay based on oxidant-induced growth arrest in *Saccharomyces cerevisiae*. FEMS Yeast Res..

[27] Marcos Rodriguez J.L., Estrella De Castro A.T., Costa A., Oliver Ruiz P.J., Fraile M.A., De La Fuente Moreno Y.N., Rodriguez J.L., Diez Garcia S.Μ., Peiro B., Cezon Munoz Ruiz E. (2004). Improved Method of Producing Lycopene through the Fermentation of Selected Strains of Blakeslea trispora, Formulations and Uses of the Lycopene Thus Obtained.

[28] Pollmann H., Breitenbach J., Sandmann G. (2017). Engineering of the carotenoid pathway in *Xanthophyllomyces dendrorhous* leading to the synthesis of zeaxanthin. Appl. Microbiol. Biotechnol..

[29] Rodriguez-Amaya D.B. (2001). A Guide to Carotenoid Analysis in Foods.

[30] Verdoes J.C., Krubasik P., Sandmann G., Van Ooyen A.J.J. (1999). Isolation and functional characterisation of a novel type of carotenoid biosynthetic gene from *Xanthophyllomyces dendrorhous*. Mol. Gen. Genet. MGG.

[31] Mantzouridou F., Naziri E., Tsimidou M.Z. (2009). Squalene versus ergosterol formation using *Saccharomyces cerevisiae*: Combined effect of oxygen supply, inoculum size, and fermentation time on yield and selectivity of the bioprocess. J. Agric. Food Chem..

[32] Naziri E., Mantzouridou F., Tsimidou M.Z. (2011). Enhanced squalene production by wild-type *Saccharomyces cerevisiae* strains using safe chemical means. J. Agric. Food Chem..

[33] Squina F.M., Mercadante A.Z. (2005). Influence of nicotine and diphenylamine on the carotenoid composition of *Rhodotorula* strains. J. Food Biochem..

[34] Guowang H., Nur F.B.I., Yimin L., Yang W., Zeng T. (2020). Naftifine inhibits pigmentation through down-regulation on expression of phytoene desaturase gene CAR1 in *Rhodotorula mucilaginosa*. Afr. J. Microbiol. Res..

[35] Vereshchagina O.A., Tereshina V.M. (2014). Trisporoids and carotenogenesis in *Blakeslea trispora*. Microbiology.

[36] Griffin D.H. (1993). Fungal Physiology.

[37] Wentzinger L.F., Bach T.J., Hartmann M.A. (2002). Inhibition of squalene synthase and squalene epoxidase in tobacco cells triggers an up-regulation of 3-hydroxy-3-methylglutaryl coenzyme a reductase. Plant Physiol..

[38] https://pubchem.ncbi.nlm.nih.gov/compound/Terbinafine#section=Toxicity.

[39] https://pubchem.ncbi.nlm.nih.gov/compound/DIPHENYLAMINE#section=Toxicity.

[40] https://pubchem.ncbi.nlm.nih.gov/compound/2-Methylimidazole#section=Toxicity.

[41] Bejarano E.R., Cerda-Olmedo E. (1989). Inhibition of phytoene dehydrogenation and activation of carotenogenesis in *Phycomyces*. Phytochemistry.

[42] Clarke I.E., De La Concha A., Murillo F.J., Sandmann G., Skone E.J., Bramley P.M. (1983). The effect of diphenylamine on carotenogenesis in *Phycomyces blakesleeanus*. Phytochemistry.

