# Supplementary material for: Uncovering the Hidden Potential of Phytoene Production by the Fungus Blakeslea trispora
Source: Foods. 2024 Sep 11;13(18):2882. doi: 10.3390/foods13182882 (PMC11431410; doi:10.3390/foods13182882)
Supplement: Supplementary file 1 [file foods-13-02882-s001.zip › foods-3153585-supplementary.pdf]

Table S1. Experimental Design for Five-Level-Six-Factor Central Composite Design.

Run	Factors					
	X_1 Diphenylamine (mg/L)	X_2 2-methyl imid-azole (mg/L)	X_3 Terbinafine (mg/L)	X_4 Addition time of inhibitor (h)	X_5 Fermentation time (h)	X_6 (+)/(-) strain ratio
**1**	20.4	176.0	0.00	33.8	122.0	0.275
**2**	20.4	176.0	45.8	67.6	122.0	0.275
**3**	20.4	176.0	45.8	33.8	122.0	0.275
**4**	11.8	102.0	65.0	19.6	76.00	0.350
**5**	20.4	176.0	45.8	33.8	122.0	0.275
**6**	29.0	250.0	65.0	48.0	76.00	0.200
**7**	11.8	250.0	65.0	48.0	76.00	0.350
**8**	11.8	250.0	65.0	48.0	168.0	0.200
**9**	0.00	176.0	45.8	33.8	122.0	0.275
**10**	29.0	102.0	26.5	19.6	76.00	0.350
**11**	11.8	250.0	65.0	19.6	76.00	0.200
**12**	20.4	176.0	45.8	33.8	122.0	0.275
**13**	20.4	176.0	45.8	33.8	122.0	0.275
**14**	11.8	250.0	26.5	19.6	168.0	0.200
**15**	29.0	102.0	65.0	19.6	168.0	0.350
**16**	20.4	176.0	45.8	0.00	122.0	0.275
**17**	11.8	250.0	26.5	19.6	76.00	0.350
**18**	20.4	176.0	45.8	33.8	122.0	0.275
**19**	20.4	176.0	45.8	33.8	122.0	0.275
**20**	20.4	176.0	91.5	33.8	122.0	0.275
**21**	11.8	102.0	26.5	19.6	168.0	0.350
**22**	11.8	102.0	26.5	48.0	76.00	0.350
**23**	29.0	102.0	26.5	19.6	168.0	0.200
**24**	29.0	250.0	26.5	48.0	76.00	0.350
**25**	29.0	102.0	26.5	48.0	76.00	0.200
**26**	40.8	176.0	45.8	33.8	122.0	0.275
**27**	11.8	250.0	26.5	48.0	168.0	0.350
**28**	11.8	250.0	65.0	19.6	168.0	0.350
**29**	20.4	176.0	45.8	33.8	12.60	0.275
**30**	29.0	102.0	65.0	48.0	168.0	0.200
**31**	20.4	0.00	45.8	33.8	122.0	0.275
**32**	20.4	352.0	45.8	33.8	122.0	0.275
**33**	20.4	176.0	45.8	33.8	122.0	0.275
**34**	20.4	176.0	45.8	33.8	122.0	0.453
**35**	11.8	102.0	26.5	48.0	168.0	0.200
**36**	11.8	250.0	26.5	48.0	76.00	0.200
**37**	11.8	102.0	65.0	48.0	168,0	0.350
**38**	11.8	102.0	65.0	19.6	168,0	0.200
**39**	20.4	176.0	45.8	33.8	122,0	0.275
**40**	29.0	250.0	26.5	48.0	168.0	0.200
**41**	29.0	102.0	65.0	48.0	76.00	0.350
**42**	11.8	102.0	65.0	48.0	76.00	0.200
**43**	29.0	102.0	65.0	19.6	76.00	0.200
**44**	29.0	102.0	26.5	48.0	168.0	0.350
**45**	29.0	250.0	65.0	19.6	168.0	0.200
**46**	29.0	250.0	26.5	19.6	76.00	0.200

**47**	29.0	250.0	26.5	19.6	168.0	0.350
**48**	29.0	250.0	65.0	48.0	168.0	0.350
**49**	29.0	250.0	65.0	19.6	76.00	0.350
**50**	20.4	176.0	45.8	33.8	231.4	0.275
**51**	20.4	176.0	45.8	33.8	122.0	0.097
**52**	11.8	102.0	26.5	19.6	76.0	0.200
**53**	20.4	176.0	45.8	33.8	122.0	0.275

Table S2. Analysis of variance of Y_{PHY}, Y_{LYC}, Y_{SQU} and Y_{ERG} obtained using the RSM model.

	Source	F value	P value
***Y_{PHY}* response** (R^2 = 0.882)			
Regression		6.890	0.000
	Linear	17.68	0.000
	Square	10.07	0.000
	Interaction	1.310	0.268
Lack of fit		3.170	0.051
***Y_{LYC}* response** (R^2 = 0.775)			
Regression		2.85	0.005
	Linear	7.38	0.000
	Square	3.68	0.009
	Interaction	0.71	0.753
Lack of fit		3.12	0.053
***Y_{ERG}* response** (R^2 = 0.783)			
Regression		2.61	0.009
	Linear	4.02	0.006
	Square	3.91	0.007
	Interaction	1.53	0.168
Lack of fit		2.95	0.062
***Y_{SQU}* response** (R^2 = 0.803)			
Regression		3.79	0.001
	Linear	1.34	0.277
	Square	5.66	0.001
	Interaction	4.02	0.001
Lack of fit		2.83	0.069

Table S3. Estimated regression coefficients and significance (p values) for phytoene content (PHY) after analysis using coded values of factors.

**Term**	**Coefficient**	**P value**
Y_{PHY} response		
Constant	1.610	0.000
Diphenylamine	0.0628	0.311
2-Methyimidazole	-0.2405	0.001
Terbinafine	0.2355	0.001
Addition Time of Inhibitors	0.4592	0.000
Fermentation Time	-0.2156	0.002
Strain Ratio (+/-)	-0.1280	0.045
Diphenylamine × Diphenylamine	0.0146	0.781
2-Methyimidazole × 2-Methyimidazole	0.1691	0.003
Terbinafine × Terbinafine	-0.0183	0.727
Addition Time of Inhibitors × Addition Time of Inhibitors	0.2733	0.000
Fermentation Time × Fermentation Time	-0.2225	0.000
Strain Ratio (+/-) × Strain Ratio (+/-)	0.0077	0.883

Diphenylamine × 2-Methyimidazole	0.0539	0.452
Diphenylamine × Terbinafine	-0.0231	0.746
Diphenylamine × Addition Time of Inhibitors	-0.0041	0.954
Diphenylamine × Fermentation Time	0.0310	0.665
Diphenylamine × Strain Ratio (+/-)	-0.0226	0.751
2-Methyimidazole × Terbinafine	0.0010	0.989
2-Methyimidazole × Addition Time of Inhibitors	0.0140	0.845
2-Methyimidazole × Fermentation Time	0.0009	0.990
2-Methyimidazole× Strain Ratio (+/-)	0.0252	0.724
Terbinafine × Addition Time of Inhibitors	0.0253	0.723
Terbinafine × Fermentation Time	-0.1083	0.138
Terbinafine × Strain Ratio (+/-)	-0.1551	0.038
Addition Time of Inhibitors × Fermentation Time	-0.2068	0.007
Addition Time of Inhibitors × Strain Ratio (+/-)	-0.0133	0.852
Fermentation Time × Strain Ratio (+/-)	0.1130	0.122

Table S4. Estimated regression coefficients and significance (p values) for lycopene content (LYC) after analysis using coded values of factors.

Term	Coefficient	P value
Y_{LYC} response		
Constant	3.851	0.000
Diphenylamine	-0.168	0.289
2-Methyimidazole	-0.232	0.147
Terbinafine	-0.213	0.182
Addition Time of Inhibitors	0.344	0.036
Fermentation Time	0.777	0.000
Strain Ratio (+/-)	-0.463	0.006
Diphenylamine × Diphenylamine	0.082	0.542
2-Methyimidazole × 2-Methyimidazole	-0.328	0.020
Terbinafine × Terbinafine	0.225	0.102
Addition Time of Inhibitors × Addition Time of Inhibitors	-0.366	0.011
Fermentation Time × Fermentation Time	-0.229	0.096
Strain Ratio (+/-) × Strain Ratio (+/-)	0.142	0.294
Diphenylamine × 2-Methyimidazole	0.118	0.519
Diphenylamine × Terbinafine	-0.092	0.616
Diphenylamine × Addition Time of Inhibitors	0.002	0.992
Diphenylamine × Fermentation Time	0.022	0.903
Diphenylamine × Strain Ratio (+/-)	0.124	0.497
2-Methyimidazole × Terbinafine	0.095	0.603
2-Methyimidazole × Addition Time of Inhibitors	0.487	0.012
2-Methyimidazole × Fermentation Time	0.027	0.882
2-Methyimidazole× Strain Ratio (+/-)	-0.025	0.889
Terbinafine × Addition Time of Inhibitors	0.037	0.838
Terbinafine × Fermentation Time	-0.095	0.604
Terbinafine × Strain Ratio (+/-)	-0.048	0.794
Addition Time of Inhibitors × Fermentation Time	0.184	0.316
Addition Time of Inhibitors × Strain Ratio (+/-)	0.028	0.877
Fermentation Time × Strain Ratio (+/-)	-0.115	0.531

Table S5. Estimated regression coefficients and significance (p values) for squalene content (SQU) after analysis using coded values of factors.

Term	Coefficient	P value
Y_{SQU} response		
Constant	0.26690	0.000
Diphenylamine	0.00678	0.323
2-Methyimidazole	0.00693	0.313
Terbinafine	0.00511	0.455
Addition Time of Inhibitors	0.01141	0.102
Fermentation Time	-0.00424	0.535
Strain Ratio (+/-)	-0.00979	0.158
Diphenylamine × Diphenylamine	-0,01139	0.059
2-Methyimidazole × 2-Methyimidazole	-0.01050	0.080
Terbinafine × Terbinafine	-0.01316	0.031
Addition Time of Inhibitors × Addition Time of Inhibitors	0.02043	0.002
Fermentation Time × Fermentation Time	-0.01492	0.016
Strain Ratio (+/-) × Strain Ratio (+/-)	0.00452	0.439
Diphenylamine × 2-Methyimidazole	-0.00906	0.258
Diphenylamine × Terbinafine	-0.00656	0.410
Diphenylamine × Addition Time of Inhibitors	0.00156	0.843
Diphenylamine × Fermentation Time	0.01531	0.062
Diphenylamine × Strain Ratio (+/-)	0.01469	0.072
2-Methyimidazole × Terbinafine	-0.01281	0.144
2-Methyimidazole × Addition Time of Inhibitors	-0.02219	0.009
2-Methyimidazole × Fermentation Time	0.00031	0.968
2-Methyimidazole× Strain Ratio (+/-)	0.01469	0.072
Terbinafine × Addition Time of Inhibitors	0.02656	0.002
Terbinafine × Fermentation Time	0.00656	0.410
Terbinafine × Strain Ratio (+/-)	-0.02281	0.007
Addition Time of Inhibitors × Fermentation Time	0.01844	0.027
Addition Time of Inhibitors × Strain Ratio (+/-)	-0.01719	0.038
Fermentation Time × Strain Ratio (+/-)	-0.01844	0.027

Table S6. Estimated regression coefficients and significance (p values) for ergosterol content (ERG) after analysis using coded values of factors.

Term	Coefficient	P value
Y_{ERG} response		
Constant	1.5826	0.000
Diphenylamine	-0.0105	0.785
2-Methyimidazole	0.0047	0.904
Terbinafine	0.0139	0.719
Addition Time of Inhibitors	0.0995	0.015
Fermentation Time	0.1522	0.001
Strain Ratio (+/-)	-0.0435	0.266
Diphenylamine × Diphenylamine	-0.0137	0.679
2-Methyimidazole × 2-Methyimidazole	-0.0367	0.272
Terbinafine × Terbinafine	0.0349	0.295
Addition Time of Inhibitors × Addition Time of Inhibitors	0.1127	0.002
Fermentation Time × Fermentation Time	-0.0879	0.012
Strain Ratio (+/-) × Strain Ratio (+/-)	-0.0040	0.904
Diphenylamine × 2-Methyimidazole	-0.0309	0.493
Diphenylamine × Terbinafine	0.0128	0.776

Diphenylamine × Addition Time of Inhibitors	0.0316	0.485
Diphenylamine × Fermentation Time	-0.0316	0.485
Diphenylamine × Strain Ratio (+/-)	0.0203	0.652
2-Methyimidazole × Terbinafine	0.0009	0.983
2-Methyimidazole × Addition Time of Inhibitors	-0.0478	0.293
2-Methyimidazole × Fermentation Time	0.0228	0.613
2-Methyimidazole× Strain Ratio (+/-)	0.0484	0.287
Terbinafine × Addition Time of Inhibitors	0.0547	0.231
Terbinafine × Fermentation Time	0.0203	0.652
Terbinafine × Strain Ratio (+/-)	-0.0178	0.692
Addition Time of Inhibitors × Fermentation Time	0.1703	0.001
Addition Time of Inhibitors × Strain Ratio (+/-)	-0.0453	0.318
Fermentation Time × Strain Ratio (+/-)	-0.0447	0.325

Table S7. Carotenoid elution order on Nucleosil C18 column, retention time (RT), relative retention time (RRT), spectral characteristics of peaks (λ_{max}) and possible identity based on the comparison with standard compounds and literature data (Pollmann et al., 2017b; Rodriguez-Amaya, 2001; Verdoes et al., 1999). Chromatographic data represent the carotenoid-rich extract from TH-treated B. trispora cells (fermentation time, 125 h).

Peak No.	RT (min)	RT (min)[a]	λ_{max} (nm)	Possible identity
1	16.45	0.64	416,440,469	neurosporene
2	18.33	0.71	439,462,491	γ-carotene
3	19.23	0.74	380,400,424	ζ-carotene
4	21.29	0.82	404,430,453	β-zeacarotene
5	23.71	0.92	(425)[b],451,478	β-carotene
6	25.83	1	(275)[b],286,(297)[b]	phytoene
7	27.38	1.06	404,429,245	7,8-dihydro-β-carotene

[a] RT of the compound peak/ RT of the phytoene peak. [b] Parenthesis indicates a shoulder.

Table S8. Sterols elution order on Accucore C18 column, retention time (RT), relative retention time (RRT), spectral characteristics of peaks (λmax) and possible identity based on the comparison with standard compounds and literature data (Naziri et al., 2011). Chromatographic data represent the UM of cellular lipids from the TH-treated B. trispora cells (fermentation time, 125 h).

Peak No.	RT (min)	RRT (min)[a]	λmax (nm)	Possible identity
1	4.8	0.75	270,280,290	ergosta-5,7,24(28)-tetraen-3β-ol
2	5.2	0.8	205	zymosterol
3	6.4	1	270,280,290	ergosterol
4	7.9	1.23	270,280,290	22,23-dihydroergosterol
5	8.2	1.28	205	lanosterol
6	12.2	1.90	208	squalene

[a] RT of the compound peak/ RT of the ergosterol peak.
